# Cloud Coverage Acts as an Amplifier for Ecological Light Pollution in Urban Ecosystems

**DOI:** 10.1371/journal.pone.0017307

**Published:** 2011-03-02

**Authors:** Christopher C. M. Kyba, Thomas Ruhtz, Jürgen Fischer, Franz Hölker

**Affiliations:** 1 Institute for Space Sciences, Freie Universität Berlin, Berlin, Germany; 2 Leibniz-Institute for Freshwater Ecology and Inland Fisheries, Berlin, Germany; Universidade de Vigo, Spain

## Abstract

The diurnal cycle of light and dark is one of the strongest environmental factors for life on Earth. Many species in both terrestrial and aquatic ecosystems use the level of ambient light to regulate their metabolism, growth, and behavior. The sky glow caused by artificial lighting from urban areas disrupts this natural cycle, and has been shown to impact the behavior of organisms, even many kilometers away from the light sources. It could be hypothesized that factors that increase the luminance of the sky amplify the degree of this “ecological light pollution”. We show that cloud coverage dramatically amplifies the sky luminance, by a factor of 10.1 for one location inside of Berlin and by a factor of 2.8 at 32 km from the city center. We also show that inside of the city overcast nights are brighter than clear rural moonlit nights, by a factor of 4.1. These results have important implications for choronobiological and chronoecological studies in urban areas, where this amplification effect has previously not been considered.

## Introduction

The ambient light level is one of the strongest factors driving animal behavior and chronobiology, evidenced by the dramatic split of most species into diurnal or nocturnal activity. It is therefore unsurprising that changes in ambient nighttime lighting result in behavioral and physiological changes for many nocturnal species [1], whether in terrestrial [2]–[4], marine [5], or freshwater [6]–[8] habitats.

With the exception of life in the deep oceans and underground, all life on Earth has evolved to live in an environment of cycles of light and dark, with a substantial proportion of the global biodiversity being nocturnal (30% of all vertebrates and 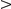
60% of all invertebrates [9]. Most organisms, humans included, have evolved molecular circadian clocks which are set by natural day/night cycles. Until the invention of artificial light, this meant that many behavioral and physiological traits were determined by the motions of the sun, the moon, the stars, and the weather (e.g. [10]–[12]).

The first lighting technology was fire, which was used expressly to modify animal behavior: fire allowed human activity to continue past sundown and frightened away human predators. Small scale urban lighting began with gas lamps, but the nighttime environment drastically changed with the widespread deployment of electric lighting in the last century. Since then, the rapid global increase of artificial light has fundamentally transformed nightscapes, both in terms of quantity, increasing several percent each year, and in quality (color spectra) [13].

Light pollution, which causes the “light dome” dome of sky glow over urban areas, is an unintended result of this electric lighting, and because of it approximately 10% of the world's population, and more than 40% of the US population, no longer view the night sky with dark adapted vision [14]. In addition to emptying the night sky of stars, it has been suggested this unwanted light may be damaging to our health [15]–[18], although this hypothesis is debated [19].

For any given individual species, the impact of artificial light may be neutral, beneficial (e.g. increased foraging), or detrimental (e.g. collisions with lighted structures [20]). In either of the latter cases this may disrupt predator-prey relationships and ecosystem functions [21], [22]. Thus, light pollution can also be considered an important driver behind the erosion of ecosystem services (e.g. pollination of plants by moths or bats, loss of biodiversity, and changes to food webs [9]). Aesthetic values, such as the visibility of the Milky Way, could be also considered a vulnerable cultural ecosystem service [23]. While the fact that artificial light affects animal behavior has been recorded since Aristotle, recognition of the potential danger posed to entire social-ecological systems by urban lighting is relatively recent [13], [24].

Sky glow occurs when light escaping upwards from a city is scattered back to the ground, through interactions with atmospheric components. On clear nights with extremely good visibility, urban sky glow is caused by the scattering of light by molecules (Rayleigh scattering). Rayleigh scattering affects blue light much more strongly than red, and is responsible for making the sky blue and the sunset red. The glow of distant cities is red for the same reason [25].

Atmospheric visibility is generally reduced due to the presence of aerosols, small particles or droplets suspended in the air that can come from natural (e.g. dust, sea salt) or artificial (e.g. soot) sources. Aerosols can impact light pollution in several ways. First, higher aerosol concentrations should amplify the sky glow (particularly on cloud free nights), as aerosols increase the chance that light is scattered back to Earth. Second, if the aerosols are absorbing in the visible band (which is typical in the case of smog), they could reduce the extent to which environmental changes (e.g. snow, or as we shall see, cloud cover) amplify light pollution, as multiply scattered light would have increased chances of absorption. Third, in the case of very short visibility, the probability of light propagating to the city limits will be reduced, and thus the horizontal extent of the sky glow outside of the city should be reduced.

Clouds are effectively thick collections of aerosols (small water droplets) that almost non-absorbing at visible wavelengths. This makes clouds very reflective [26], [27], and therefore we expect them to amplify sky glow. In the case of optically thick clouds, if we consider only the upward and downward propagation of light (as in the so-called two-stream approximation), then, to first order, the cloud bottom can be thought of as a two-sided, white (Lambertian) boundary, which diffusely reflects sun, moon, or city light back towards the hemisphere from which it came. While this analogy is clearly an oversimplification (e.g. one can usually see quite well outdoors in the daytime even under thick clouds), it is useful for gaining a “feel” for how clouds interact with light pollution. In the particular case of an observer under optically thick clouds and inside of a large city (where the cloud bottom is much closer to the observer than is the edge of the city), the model of the cloud bottom as a Lambertian surface is probably a reasonably good approximation.

**Figure 1:**
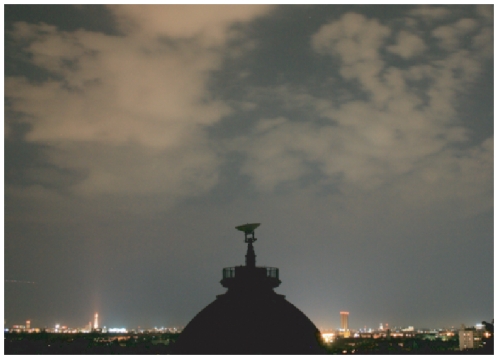
Photograph showing the amplification effect that clouds have on the sky glow. Inside of cities clouds appear as bright objects on a dark sky. In natural environments, clouds look more like the tower in the photo: dark silhouettes against a star-lit sky. Photo: C Kyba.

This redirection of light back towards the ground gives rise to the effect shown in Figure 1, that while in pristine environments clouds appear as dark objects on the star filled sky, in cities clouds appear as bright objects on a dark background, nearly devoid of stars. While this phenomena has been qualitatively observed by many people, we believe that this work represents the first systematic and quantitative study of this effect presented in the scientific literature. The reasons a similar study by [28] did not observe this effect are considered in the discussion section. Measurements of the increase of light due to cloud coverage were shown in [29] and in a poster by Posch, Hollan, Kerschbaum, and Bleha presented at the Cancer and Rhythm conference, Graz Austria, 2004, but in both cases only for single nights.

While an atmospheric model of clouds as white surfaces and “Rayleigh scattering only” skies is sufficient to qualitatively discuss the results of this paper, we should note that quantitative modeling of light pollution requires much more attention to detail. For an observer on the ground, the radiance of the sky observed in any given direction depends upon a host of variables, including the wavelength of the light in question, the makeup of the atmosphere, the distribution of city lights on the ground, the topography of the city, and the observer's position within it. In the next two paragraphs we point to more detailed references, which together describe each of the components needed to fully characterize the sky glow produced by a city.

The scattering and absorption of light in the atmosphere is of central importance to climate science, and has thus been described in detail elsewhere (see e.g. [27], [30]). Modeling the interaction of light pollution with clouds requires understanding of the optical properties of the cloud, in particular the cloud optical thickness (a description of the probability that light interacts with water droplets in the cloud), the single scatter albedo (the propensity of photons to be scattered rather than absorbed), and the asymmetry parameter (the relative proportion of photons that are scattered forward rather than backward) [27]. A detailed discussion of cloud reflectance can be seen in e.g. [26].

In most cases, atmospheric scientists focus discussion on the interaction of sunlight with the atmosphere. Light pollution, however, is very different from sunlight in that the angular distribution of upward traveling light depends strongly on position, and the spectral distribution depends very strongly on local factors (i.e. what types of lamps are in common use). An evaluation the combined luminance of all of the sources of light a single city is given in [31], a comprehensive review of the spectrum of different lamp types is given in [32], and discussion of the geometry of light pollution, and sky maps showing the sky radiance caused by single or multiple lamps is given in [33].

Historically, light pollution research and advocacy has been undertaken by astronomers, who justifiably have little interest in cloudy nights. In the first serious model of light pollution [34], only the case of clear skies was considered, and with some exceptions (e.g. [33]), models and measurements generally consider only cloud-free conditions [14], [35]–[38].

We expect the presence of clouds to significantly brighten urban skies, and to amplify the degree of ecological light pollution. We aim to show that in studying the impact of sky glow on ecology, health, or interruption of circadian rhythm, it is essential that cloud coverage be taken into account. In performing our analysis, we also expect to show that the level of light pollution in Berlin is ecologically relevant (meeting or exceeding the light levels produced by the moon), and finally that the total light produced by Berlin decreases as the night progresses.

## Materials and Methods

The main goal of this paper is to measure how cloud coverage affects sky brightness in an urban environment. This measurement is referred to as the “cloud analysis”. In order to allow for comparisons to the sky brightness typical of natural environments, we also study how the elevation of the moon above the horizon affects sky brightness. This is called the “moon analysis”.

**Figure 2:**
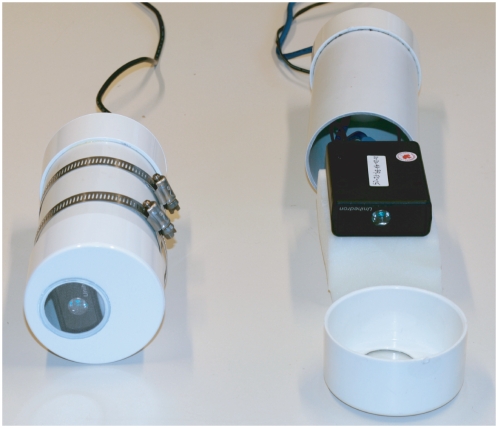
Photograph showing the Sky Quality Meter installed in its protective housing (SQM-LU left), along with an expanded view (SQM-LE right). The housing at left is shown with the two included hose clamps that allow easy attachment to a stake or pole. The USB version (left) requires only one cable, but at the cost of shorter cable runs and the internal heating provided by the Ethernet version (right).

Our night sky brightness data were taken using the “Sky Quality Meter” (SQM) produced by Unihedron (Grimsby, Canada), shown in Figure 2. The SQM measures luminance (surface brightness) for a patch of the sky, in units of magnitudes per square arc second (mag/arcsec^2^). The photosensitive element of the meter is a silicon photodiode (TAOS TSL237S light-to-frequency converter), which responds to light with wavelengths in the range of 320 to 1050 nm, with a peak at about 680 nm. The photodiode is covered by a HOYA CM-500 filter, which reduces the wavelength response to 320 to 720 nm, in order to provide better agreement with the wavelength response of human night vision. The response of the TSL237S has a small, stable, temperature dependence, so the SQM contains an internal temperature sensor which is used by the SQM software to provide compensation (i.e. the results reported by the SQM should have no temperature dependence over the range −25 to 70 degrees Celsius).

Unihedron produces several different models of the SQM, which are differentiated by their method of data readout and by their field-of-view (FOV). The SQM is available as a hand-held device with a digital display, or as a continuous measurement device using either a USB or Ethernet connection. We made use of Ethernet enabled SQMs, as Ethernet allows longer cable runs than USB. The field-of-view is determined by the presence (SQM-LE) or absence (SQM-E) of a focusing lens, which reduces the FOV from a wide angle to a small patch of the sky. The half-width at half-maximum has been measured to be 42 and 10 for the SQM-E and SQM-LE respectively [39]. For the measurements reported in this paper, we made use of one SQM-LE and two SQM-E devices.

The SQM reports the sky brightness in units of magnitudes per square arcsecond (mag/arcsec^2^), a logarithmic unit in use by the astronomy community. The scale is defined so that an *increase* of 5 in mag/arcsec^2^ corresponds to a factor of 100 *decrease* in luminance. It is possible to approximately convert mag/arcsec^2^ into nit (cd/m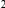) using the formula: cd/m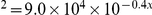, where 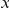 is the luminance in mag/arcsec^2^. (This equation was provided to us by Unihedron, and originates from the webpage of Paul Schlyter: www.stjarnhimlen.se/comp/radfaq.html) This conversion, however, contains an implicit assumption about the wavelength distribution of star light, which we can neither assume to be the same as light pollution, nor the same for both clear and cloudy conditions. A general conversion from mag/arcsec^2^ to lux is not possible, as converting luminance measurements to illuminance measurements requires making an assumption about the angular distribution of the sky brightness intensity, which we expect to change in the presence of clouds. The SQMs have a quoted systematic uncertainty of 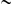
10% (0.10 mag/arcsec^2^).

The Ethernet enabled SQMs were installed at three locations: our measurement tower at the Institute for Space Sciences at the Freie Universität (52.4577

N, 13.3107

E), at the Leibniz-Institut of Freshwater Ecology and Inland Fisheries (52.4487

N, 13.6513

E), and on an island of the Spree river outside of the city (52.3681

N, 13.8049

E). The locations are approximately 10, 18, and 32 km from the center of Berlin, and can be classified as urban, suburban, and rural respectively. In order to protect the devices from rain and snow, the SQMs were installed in a protective housing produced by Unihedron. The housing consists of a short length of 3" PVC pipe fitted on the top and bottom with 3" PVC endcaps. The bottom endcap has a hole drilled in it to allow for entry of cables and to allow moisture to escape, and the top endcap has a hole to allow a window for observations. This window is covered with a glass top which is glued to the surface of the endcap. The attenuation of the glass cover has been measured to be 0.11 mag/arcsec^2^ [40], and to correct for this effect we subtract this amount from the readings reported by the device. The internal web server of the SQM-LE produces enough heat to quickly melt snow and evaporate water from the glass surface.

The data were read out from the SQMs using a custom developed Perl script, partially based on sample code provided by the manufacturer. The devices were polled approximately once per second, and whenever the readout value changed, the time and sky brightness measurement were recorded to a file. These values were then averaged by our analysis program to create a minute-by-minute dataset. Despite the logarithmic scale, we directly averaged the measurements in mag/arcsec^2^, as we expect measurement differences at very short time scales are more likely to be due to the device electronics rather than a physical change in the sky brightness.

Our cloud coverage figures were taken from synoptic measurements at a manned weather station (Berlin-Dahlem, World Meteorological Index 10381) located adjacent to our measurement location at the Freie Universität. The SYNOP data were retrieved from the OGIMET website, http://www.ogimet.com. In synoptic observations cloud coverage is reported in “oktas”, which represent the fraction of the sky obscured by cloud in eighth's. Zero oktas corresponds to a cloud-free sky, while eight oktas corresponds to completely overcast conditions. The synop data were reported hourly, so the maximum time difference between any sky brightness measurement and the most recent cloud observation was 30 minutes. Berlin has several synop stations, and we have verified that using cloud data from a different station (e.g. closer to the rural site) has only a minor impact on the results. Because we are most interested in what is happening at the urban location we used the data from the adjacent weather station.

In natural ecosystems, the moon is the brightest source of light at night. The relationship between the intensity of moonlight (both direct and scattered) and the moon's position parameters (distance from Earth, phase, elevation above the horizon, and time of year) is computationally very complex (see e.g. the simulation presented in [28]). We eliminated the need to compensate for moon lighting in the cloud analysis by simply considering only moonless nights. To do this, it was necessary to calculate the position of the moon for each data point. This was accomplished using the “Astro::Coord::ECI” and “Astro::Coord::ECI::Moon” open source Perl scripts (v0.033), which were developed by Thomas R. Wyant, and are freely distributed by the Comprehensive Perl Archive Network (http://search.cpan.org/ 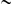
wyant/Astro-satpass−0.033/). The algorithm is based upon calculations in [41], and has a quoted moon position uncertainty of 10 seconds of arc in latitude.

The moon positioning algorithm was initialized using the longitude, latitude, and elevation (91 m) of the measurement tower at the Freie Universität. The moon position was calculated at the 30 second mark of each minute, matching the median time of our sky brightness measurements. We define our data to be “moonless” when the moon's true position (i.e. ignoring any effects of refraction in the atmosphere) is 2 or more below the horizon.

The sky brightness data for the cloud analysis were taken during the period from April 22 to September 21, 2010, using wide field-of-view SQM-Es at our urban and rural measurement stations (10 and 32 km from the city center, respectively). Within this time span the data from six nights were rejected due to failures in the data acquisition chain (e.g. from a power interruption). The summer air in Berlin is relatively clean, and visibilities of 30–40 km were typical during this study. Because we expected the total amount of light produced by the city to decrease as the night progressed (from decreased auto, residential, and advertising lighting), we only considered data taken during the same time window each night. This considerably restricts the size of our dataset, but reduces the possibility of introducing systematic bias or larger variation into the observed sky glow.

The optimal duration of the data taking window depends upon the analysis that one wishes to pursue. For the cloud analysis our goal was to include as many different cloud coverage values as possible; getting a “snapshot” of the sky brightness at the same time as the cloud coverage measurement was taken, and for a variety of weather conditions, was more important than sampling unchanging skies over several hours. The large size of weather systems means that overcast or clear conditions often persist for several days, and for this reason we wished to use data from as many nights as possible. Due to the extremely short duration of the night at the time of the summer solstice in Berlin, this restricted us to using only data taken between 12:45 am and 1:15 am local time (UTC+2, Central European Summer Time). Berlin is near the center of its time zone, so the moment of “true” local midnight occurred during this half-hour period for each night in our dataset.

In the case of our moon analysis we were less concerned with including as many individual nights as possible. Instead, we preferred to use a longer time interval each night, which allowed the moon to move through a substantial range of elevation above the horizon each night. For this reason we only used data taken at least three weeks away from the summer solstice (i.e. April 22-May 30, and July 13-September 21). This allowed us to use a wider time window than that used in the cloud analysis, from 12:00 am to 2:00 am. To avoid the possible influence of clouds, we only included data for which the cloud coverage in the two synop reports nearest to the sky brightness measurement was 0 or 1 okta.

The computer reading out the data at the Freie Universität has access to an Internet connection, which allowed timing to be maintained to better than second accuracy throughout the data taking period. The computer collecting data at the remote location, however, was located in a non-climate controlled container, and experienced clock drift. This computer's time was periodically corrected manually, at intervals ranging from 5 to 38 days. When these corrections were made, the total drift since the last correction was noted. This allowed us to remove the linear portion of the clock drift in software, and pass the corrected data to our analysis program after the data was collected. Over the entire period of data taking, the average clock drift was +12.9 seconds/day, and we do not expect that the maximum deviation from true time at any period in our dataset was more than 5 minutes.

## Results

**Figure 3:**
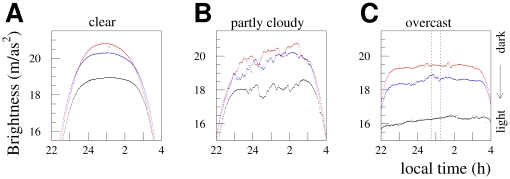
Examples of the sky brightness (in mag/arcsec^**2**^) observed for different cloud conditions and at different locations. The minute by minute data for individual clear (A, June 4–5), partly cloudy (B, May 20–21), and overcast (C, May 13–14) nights at each of our rural (red), suburban (blue), and urban (black) measurement stations is shown. Larger values of mag/arcsec^2^ indicate darker skies. The unit is logarithmic, with a 2.5 increase in mag/arcsec^2^ corresponding to a sky that is 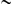
10 times as dark. The dotted lines in the plot at right show the time window used in the cloud analysis.

The sky brightness values recorded on three representative nights (clear, partly cloudy, and overcast) at our three measurement locations are shown in Figure 3. In all weather conditions, the rural site was darkest (largest value of mag/arcsec^2^) and the urban site was brightest. The plot at left shows the data for the clear (0–1 oktas) night of June 4–5, 2010, during which the half full moon rose at 1:21 am. The middle plot shows data for May 20–21, which was partly cloudy (3–4 oktas) until 3 am, when the sky cleared (to 1 okta). The right hand plot shows the data for May 13–14, which was overcast (8 oktas) the entire evening. A dotted line is drawn in the right hand panel to show the portion of the data from that night that contributes to the cloud analysis.

At midnight on the clear night in the left hand frame of Figure 3, the sky brightness at the rural site was on average about 1.85 mag/arcsec^2^ darker than the urban site (2.4 mcd/m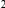 compared to 0.43 mcd/m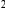, 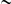
1/5 the luminance), while on the overcast night it was 3.15 mag/arcsec^2^ darker (26 mcd/m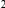 compared to 1.4 mcd/m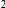, 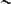
1/20 the luminance). It is immediately apparent from these plots that the sky glow exhibits a strong urban
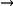
rural gradient, and that clouds have a very significant impact on urban sky brightness. Note that the suburban data were taken with a narrow FOV SQM-LE, which we found tends to record darker values for clear and partly cloudy conditions. We included the suburban data in Figure 3 to emphasize the urban
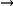
rural transition, but we do not use the data from that location in our cloud or moon analyses.

While we would in principle prefer to have equivalent statistics for each level of cloudiness, in practice we must make use of the conditions that nature provides. Table 1 shows the number of nights in the dataset for which each degree of cloudiness was observed at 1 am. The table also shows the effective number of nights available for the cloud analysis. Fractional values occur because of occasional data loss, and because of nights during which the moon rose or set during the 30 minute analysis period. Clear or overcast conditions occurred much more frequently than partly cloudy (2–6 oktas) skies.

**Table 1:** Frequency of cloud coverage conditions over the course of data taking.

Oktas	0	1	2	3	4	5	6	7	8
Total Nights	21.5	26.5	7	12.5	7	13	10	23	26
Moonless Nights	11.9	10.6	3	7	0	5	6	9.2	13.6

For each value of cloud coverage (0 is clear, 8 is overcast), the number of nights in the observation period is shown along with the effective number of nights that the moon was at least 2 below the horizon between 12:45 and 1:15 am. Fractional values occur due to occasional data loss due to power outages, and to nights during which the moon rose.

**Figure 4:**
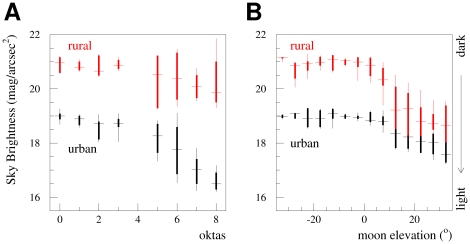
Profile histograms of the sky brightness data. Panel A shows the sky brightness observed as a function of cloud coverage. The bars show the 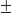 1 and 2 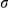 spread of the data. Panel B shows the sky brightness as a function of moon elevation for clear (0–1 okta) nights. Larger values of mag/arcsec^2^ indicate darker nights.

Our results for the cloud analysis using the full dataset are shown in the left panel of Figure 4, and numerically in Table 2. In the figure, the upper set of points represent the data at the rural location, while the lower set were taken inside of the city. For each value of cloudiness (in oktas) the median sky brightness observed is shown with a horizontal line. The variation in the observed data is shown by the thick and thin lines, which cover the 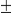
1 and 2 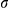 bands (containing 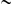 68% and 95% of the observed data, respectively). The large separation between the distributions for clear and cloudy conditions at the urban site refutes the null hypothesis (i.e. that clouds do not amplify urban sky glow) with certainty.

**Table 2:** Amplification factor of clouds.

Oktas	0	1	2	3	4	5	6	7	8
Rural (mag/arcsec^2^)	21.0	20.8	20.7	20.9	/	20.5	20.4	20.1	19.9
Urban (mag/arcsec^2^)	19.0	18.9	18.7	18.7	/	18.3	17.8	17.0	16.5
Rural amplification	1	1.2	1.3	1.1	/	1.5	1.7	2.3	2.8
Urban amplification	1	1.1	1.3	1.3	/	2.0	3.1	6.1	10.1

For each value of cloud coverage (0 is clear, 8 is overcast), the median observed sky brightness over the course of data taking is shown in mag/arcsec^2^. These data were used to calculate a sky brightness amplification factor for each level of cloudiness (relative to clear skies). Under clear conditions urban skies were 6.1 times brighter than at the rural site.

**Figure 5:**
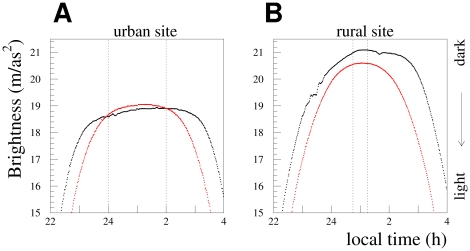
Nights are dramatically shorter around the time of the summer solstice. The minute by minute sky brightness data (in mag/arcsec^2^) for the night of June 16–17 (red) is compared to July 20–21 (black) at our urban (A), and rural (B) locations. In the left plot the dotted lines indicate the time window used in the moon analysis, and in the right plot the time window used in the cloud analysis. Due to the shortening days we reject data taken within three weeks of the summer solstice from our moon analysis. The curve for July 20–21 at the rural site appears lopsided because the moon set shortly before 1am.

We found that on the clearest nights around the time of the solstice, the sky at the rural location doesn't appear to get quite as dark as it might on an equivalent night in the spring or fall. As is shown in Figure 5, on these nights the 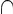 shaped pattern of the sky darkening and then brightening doesn't include the typical broad plateau. However, due to both our narrow time window of 15 minutes around 1:00 am, the large number of clear nights, and the marked difference between the urban and rural measurements, the impact of this effect is a minor increase in the spread of the data for the darkest nights. As a test, we tried selecting data within 15 minutes of 1:08 am (which is a better approximation of local midnight), and found that this had a negligible impact.

The results of the moon analysis are shown in the right hand panel of Figure 4. The data are grouped in bins of 5 of moon elevation above the horizon, and the bars show the 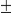
1 and 2 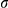 bands, as in the plot at left. Negative values of elevation indicate that the moon was below the horizon, and are shown in individual bins as a consistency demonstration.

**Figure 6:**
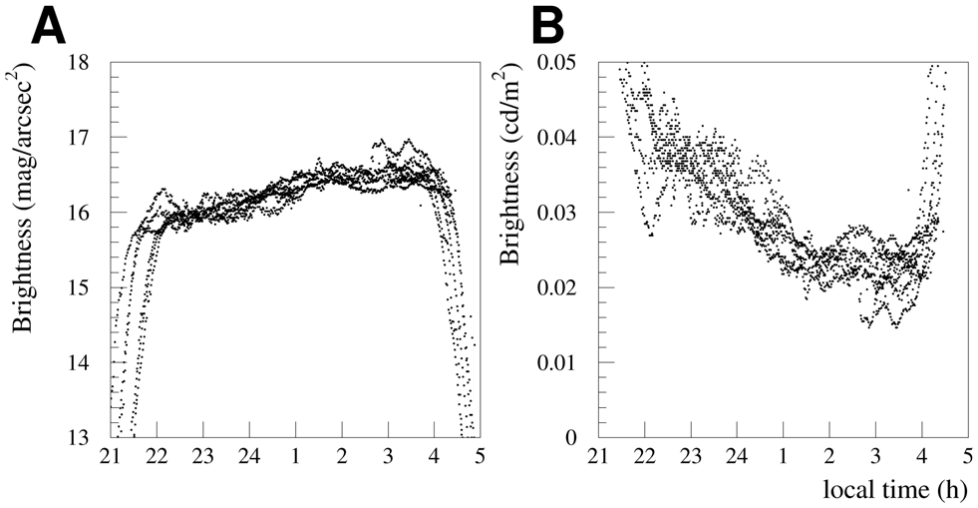
The sky brightness measured at the urban location is shown against local time for overcast skies in the April 26 - May 15 period. Data were included if the cloudiness was reported as 8 oktas in both the hourly report before and after the data was taken. Panel A shows the minute-by-minute data in the usual logarithmic scale (mag/arcsec^2^), panel B shows the same data on a linear scale, using the approximate conversion to cd/m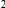. The data shown were taken during the nights of April 26, May 2–3, May 6–7, May 9–10, May 11, and May 13–15, 2010.

As discussed in the Materials and Methods section, the analysis uses only a small portion of the data from each night because the total amount of light produced by the city is expected to change as the night progresses. We tested this hypothesis by selecting a small number of nights with completely overcast skies. In order to guarantee overcast skies, data were only included if the cloudiness was 8 oktas in both of the adjacent hourly synop reports. Figure 6 shows how the sky glow over the Freie Universität changed during nights between April 26 and May 15. The left hand plot shows the data in mag/arcsec^2^, the right hand plot shows the same data on a linear scale, using the approximate conversion to cd/m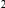 (nit). Over the course of the night the sky brightness decreased from 15.95 to 16.55 mag/arcsec^2^, a decrease in luminance of approximately 40%.

The data on which these results are based is provided in supplemental File S1. The table's contents are: the date, time of observation in “hours after midnight” in the GMT+1 time zone (i.e. +0.5 is 12:30:00 am, and −0.0083 is 11:59:30 pm), the sky brightness value observed at the urban and rural sites (in mag/arcsec^2^), the cloud coverage from the most recent SYNOP report in okta, the difference in oktas between the two adjacent SYNOP reports, the cloud base (an integer code number as per the SYNOP specifications, see e.g. http://weather.unisys.com/wxp/Appendices/Formats/SYNOP.html), the visibility (in meters), and finally the elevation (in degrees), illuminated fraction, and distance (in km) of the moon.

## Discussion

Using two SQMs, we studied changes in the sky brightness of Berlin in a rural and urban location over a period of 152 calendar days. The degree to which Berlin's skies are polluted by light can be demonstrated by comparing the sky brightness measured here with that measured in a more natural setting. In a recent study of sky brightness at the Zselic Landscape Protection Area in Hungary (an International Dark-sky Park), the darkest measurements obtained on clear moonless nights using an SQM were 21.5–21.6 mag/arcsec^2^ [38]. The very darkest observations for clear moonless nights in Berlin were 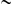
21.2 mag/arcsec^2^ at our rural location and 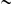
19.3 mag/arcsec^2^ at our urban location, a luminance greater by 38% and 690%, respectively. Typical nights at both locations, however, were far brighter even than this.

The left hand plot of Figure 4 demonstrates the significant degree with which clouds amplify the impact of light pollution. The data show a strong dependence on the cloudiness level, with very rapid brightening as the sky becomes fully overcast. The mean observed sky brightness for fully overcast skies at our urban measuring station was 16.5 mag/arcsec^2^, a luminance approximately 10600% brighter than that observed for dark nights at the dark-sky park in Hungary.

We can see that this sky brightening is ecologically relevant by comparing the brightness at the urban station to the brightness observed on moonlit, cloud free nights at our rural station. The two panels of Figure 4 show that regardless of weather conditions, the night sky of Berlin is almost always as bright as that naturally experienced during a high elevation summer moon. (Although it should be kept in mind that the SQM-E effectively measures the integral amount of light incident on a plane parallel to the ground. The angular distributions of sky glow and direct moonlight, and therefore an organism's visual experience of the environment under the two, are very different.) This means that for light avoiding organisms that moderate their behavior in the presence of moonlight, for example zooplankton in a lake system [42], the light pollution from Berlin is expected to be a considerable stressor. It has been previously shown, in lake food webs, that light mediated diurnal vertical migrations of zooplankton may be suppressed, decreasing the grazing pressure on phytoplankton [7], [43].

The amplification of sky glow by clouds surely amplifies this stressor, since we observed that the sky glow typical on overcast nights within Berlin was 4.1 times as bright as that observed outside the city on clear nights with a high elevation moon. In pristine ecosystems at a similar latitude to Berlin, a sky glow brighter than 19 mag/arcsec^2^ is likely only experienced for several hours on a few nights each summer, namely on cloud free nights when the moon happens to be high in the sky. This “worst case scenario” for some zooplankton species in their natural environment represents almost the most favorable conditions they can ever face in the urban waterways of Berlin. While it can be expected that some species will be genetically capable of adapting their behavior, physiology, growth, and reproduction to live in or take advantage of unnaturally lit environments, other species will not, and at least some light-sensitive species and genotypes will be lost in the long term [9].

The “error bars” shown in Figure 4 are not uncertainties, but rather represent the spread of the observed data. For the data in the cloud analysis there are three sources of variation. First, during a single night, changes in the local cloud coverage (i.e. the positions of clear and cloudy patches of the sky relative to the SQM) lead to changes in the measured sky brightness, in part due to the angular response of the SQM. This was shown in Figure 3. While only data taken within 15 minutes of the synoptical observation we considered, in some cases the cloud coverage changes during this time. Second, “oktas” are a relatively crudely spaced measure, and are determined by human observers, each of whom might have a slightly different idea of where the cutoff lies between, say, 3–4 oktas. Third, from night to night the baseline value for a given number of oktas is expected to be different, due to differences in the environmental conditions: cloud type (i.e. the cloud height, optical thickness, single scatter albedo, and asymmetry parameter), surface albedo, visibility, and atmospheric aerosol content. It is this second source of variation that gives rise to some of the “lopsided” distributions, where the upward and downward lengths of the 1 or 2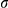 bars differ considerably in length. For example, the large upward tail on the rural 8 okta measurement in Figure 4 is due to a night with exceptionally low clouds (100–200 meter ceiling). Finally, in the case of the rural data, the cloud condition at the rural site may be slightly different than at the urban site, where the synoptical observation was made.

We believe the largest source of the night to night variation, and the reason for the steep increase in brightness with cloud level at 4–5 oktas, is changes in cloud type and thickness. Scattering from aerosols is strongly forward peaked, so while light may be deflected as it propagates through a thin cloud it is not particularly likely to be scattered back towards the ground. Thick clouds on the other hand, are expected to be very efficient at scattering light back down to ground level, as the photons must undergo many scattering events before leaving the cloud top. The hypothesis that the night to night variation is due to changes in cloud type could be easily tested by repeating this experiment in a location that has access to continuous LIDAR measurement of cloud properties.

We have demonstrated that in Berlin, and presumably in urban areas in general, cloud coverage has a strong amplification effect on light pollution. Due to this amplification, the luminance of the night sky in Berlin is 10.1 times brighter on overcast nights than on clear moonless nights, and 4.1 times brighter than that observed at our rural location on the brightest clear nights with a high elevation moon. Since many organisms are known to modify their behavior in the presence of moonlight, and because of the high frequency of overcast conditions, the cloud amplification effect has strong implications for the ecology of urban areas. The influence of cities extends over large areas: at 32 km from the city center the impact of clouds was still to brighten (by a factor of 2.8), rather than to darken, the night sky.

In contrast to the results reported here, a similar study undertaken in Hong Kong as part of a Master's thesis did not find a dependence of the night sky brightness on cloud coverage [28]. Although there were several methodological differences between that study and the present work, we believe that the primary reason for the different conclusions is that the studies were taken under completely different environmental conditions. The horizontal visibility measured by the synop station in Hong Kong was typically between 4 and 12 km. This is far shorter than that reported in Berlin, which was in almost all cases 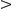
10 km. A second important difference is that the data presented in [28] are for a site 15–20 km away from Hong Kong itself, a very large distance compared to the typical visibility. It may be that an examination of the data taken within Hong Kong itself would reveal a stronger relationship between cloud coverage and sky brightness. We agree with the suggestion in [44], that duplication of this study in other cities could help to elucidate the interaction between visibility, aerosols, clouds, and sky brightness, particularly if the site has access to LIDAR data.

The recent development of convenient sky brightness meters (both the Sky Quality Meter and the International Year of Astronomy Lightmeter) has made the continuous monitoring of ecological light pollution simple. The long term deployment of these devices by light pollution researchers in cities and dark sky parks, and by ecologists and physiologists in their research environments, will allow for both a quantitative understanding of the difference in night lighting across social-ecological systems, and for systematic, high precision, ground based tracking of year-to-year changes in sky brightness.

The well known map of world light pollution [14] includes by necessity only data from clear nights. Our analysis indicates that it is very important that biological conclusions based upon those results (e.g. [16]) consider the potential role that weather plays in enhancing the brightness of urban areas. Additionally, researchers performing *in situ* experiments in or near urban areas in which the presence or absence of the moon is known to affect the result (e.g. insect catches, [4], [45]) should be aware that clouds and aerosols may play a larger role than the moon in determining ambient lighting.

It may be the case that the regional frequency of overcast nights is more important than population density in determining the threat posed to urban ecosystems by light pollution. By extending this analysis to include cities and towns of varying size, different regions, rural areas, and dark sky parks, we could test if this is the case. The development of a global dataset of continuous measurements from sky brightness meters would allow for rigorous evaluation of the results of [14], would provide strong constraints for verifying light pollution models, and would be beneficial to ecologists and light pollution researchers everywhere. We encourage anyone interested in participating in such a measurement to contact us.

## Supporting Information

File S1 (TXT)
